# Low Depth Epigenetic Mapping of Maturation Versus Retrodifferentiation in HepaRG Cells

**DOI:** 10.3390/epigenomes10020036

**Published:** 2026-06-02

**Authors:** Hector Hernandez-Vargas, Kilian Petitjean, Marie-Pierre Lambert, Yoann Daniel, Isabelle Chemin, Anne Corlu, Chloe Goldsmith

**Affiliations:** 1 Genomics Consulting, 69500 Bron, France; marie-pierre.lambert@genomicsconsulting.eu; 2 Institut NuMeCan (Nutrition, Metabolisms and Cancer), Inserm UMR_S 1317, Inrae UMR_A 1341, Université de Rennes, 35033 Rennes, France; kpetitjean@health.ucsd.edu (K.P.); yoanndan@usc.edu (Y.D.); anne.corlu@univ-rennes.fr (A.C.); 3 INSERM Unité Mixte De Recherche 1350, PaThLiv IHU EVEREST, Université Claude-Bernard Lyon 1, 69003 Lyon, France; isabelle.chemin@inserm.fr; 4 School of Science, Western Sydney University, Paramatta, NSW 2150, Australia

**Keywords:** DNA methylation, long read sequencing, cell fate decisions

## Abstract

**Background:** Long-read, single-CpG-resolution sequencing is redefining the information-to-depth ratio in epigenomics. While conventional methylome analysis often requires high coverage, we propose a scalable pipeline designed to extract high-density regulatory logic from shallow sequencing data. **Methods:** By utilizing the progenitor-like HepaRG cell line as a model for liver plasticity, we validated this framework across two divergent developmental trajectories: hepatic maturation and sphere-induced retrodifferentiation. Our technical approach combines CpG-centric enrichment and regional methylation aggregation to reconstruct regulatory landscapes from sparse data. Using long-read Nanopore sequencing, we mapped the dynamics of 5-methylcytosine (5mC) and 5-hydroxymethylcytosine (5hmC). **Results:** Our pipeline revealed that these trajectories are not inverse processes but engage distinct epigenetic strategies. Hepatic maturation is characterized by the accumulation of 5hmC that partially targets repressive heterochromatin (H3K9me3, H4K20me3) and pioneer factors such as FOXA2. In contrast, retrodifferentiation increases 5mC, potentially silencing adult regulators such as HNF1A via Polycomb-associated networks. In addition, aggregation-based analysis can distinguish widespread focal perturbations from a restricted subset of transcription factors that translate epigenetic changes into regional accessibility. **Conclusions:** This study provides a scalable computational framework for investigating cellular fate transitions, proving that high-value epigenetic insights are attainable even at reduced sequencing depths.

## 1. Introduction

The liver exhibits a remarkable degree of cellular plasticity, where mature hepatocytes can regenerate tissue or, under chronic stress, undergo retrodifferentiation toward progenitor-like states [1]. This bidirectional potential is a hallmark of hepatocellular carcinoma (HCC), where cancer stem cell-like populations drive chemoresistance and relapse [2,3]. The human HepaRG cell line serves as a robust in vitro model for this hierarchy, capable of maturation into hepatocyte-like cells or sphere-induced reversion to a primitive state [4,5]. While previous studies have documented the role of DNA modifications [6,7] and the metabolic rewiring of these transitions, the underlying epigenetic logic remains only partially resolved [8].

The advent of long-read Nanopore sequencing has revolutionized the field by enabling the simultaneous, native detection of 5-methylcytosine (5mC) and 5-hydroxymethylcytosine (5hmC) without the DNA degradation associated with bisulfite conversion. However, modification-aware sequencing at shallow coverage remains a significant barrier. Low-depth data often results in sparse genome-wide CpG representation, which impairs conventional Differentially Methylated Region (DMR) callers that rely on high-density contiguous reads. In these contexts, critical regulatory remodeling events are frequently lost to noise or insufficient statistical power.

To extract maximum regulatory information from restricted sequencing depth, we implemented a three-tiered analytical pipeline designed to amplify signal from sparse data:

- We first identified differentially methylated loci (DMLs) using the DSS package [9], which uses a beta-binomial model that borrows information from neighboring CpG sites, increasing statistical power in low-coverage contexts.
- We then used the KnowYourCG (KYCG) [10] supervised approach to study functional enrichment of these DMLs across curated transcription factor binding sites (TFBS) and chromatin annotations. This allows for regulatory mapping even when genome-wide coverage is non-contiguous.
- Finally, we applied Methylation-Based Inference of Regulatory Activity (MIRA) [11,12] to aggregate sparse signals into coherent regional profiles. This step determines if focal CpG-level perturbations translate into broad, functional shifts in chromatin accessibility.

Using this integrative strategy, we profiled 5mC and 5hmC dynamics in HepaRG progenitors grown as 2D differentiated cells or as 3D retrodifferentiated spheres. We hypothesized that our pipeline would reveal distinct “epigenetic signatures” for each state. By prioritizing aggregation-based inference over traditional high-depth requirements, this study delineates the asymmetric logic of hepatic plasticity and provides a scalable, cost-effective template for high-resolution epigenomic discovery.

## 2. Results

### 2.1. Global Imbalance in 5mC and 5hmC in Diff vs. Sphere HepaRG Culture

**Figure 1:**
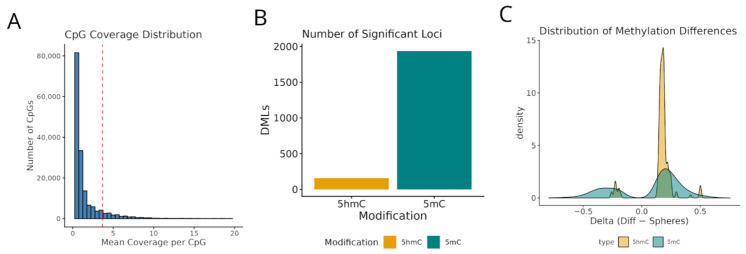
Nanopore sequencing CpG coverage and distribution of differentially methylated sites. DNA was sequenced on the Nanopore MinION; reads were basecalled with Guppy; methylation was called with Remora, and differential methylation was determined with DSS. (**A**) Mean coverage (reads per sample) of CpG sites selected for testing. The red dashed vertical line indicates the overall mean coverage across all analyzed CpG sites. (**B**) Number of differentially methylated loci with FDR-adjusted *p* value below 0.05 (DMLs) at the 5mC (green) and 5hmC (yellow) level. (**C**) Distribution of methylation differences showing the direction and magnitude of the differences in the x-axis (Diff vs. Spheres).

HepaRG cells were cultured under differentiation or retrodifferentiation conditions, as described in Methods, and will be referred to as Diff or Spheres, respectively. We studied differential methylation at the single-site level using those CpG sites that were represented (1x in at least two replicates) in both experimental conditions at a minimum coverage (Figure 1A). Such coverage filtering yielded a testing universe of approximately 171,000 CpGs, with the final number of 5mC DMLs at ~2 k CpG sites when comparing Diff vs. Spheres (Figure 1B and Table S1).

Compared to 1936 5mC DMLs, only 159 5hmC DMLs were identified (Figure 1C). Unless stated otherwise, hypo- and hyper-methylation are defined, respectively, as a directional loss or gain, relative to the Spheres condition. At the 5mC level, more DMLs were found hypermethylated (n = 1328) than hypomethylated (n = 608) in Diff relative to Sphere culture conditions (Figure 1C and Table S1). However, the distribution of 5hmC DMLs was even more unbalanced, with 147 CpGs found hypermethylated (92%) in Diff out of 159 DMLs in total (Figure 1C and Table S2). For those few CpG sites with concomitant significant changes in 5mC and 5hmC (n = 6), there was an inverse correlation between 5mC and 5hmC (Tables S1 and S2).

Overall, the comparison between differentiation and sphere conditions revealed significant 5mC remodeling and limited but unbalanced 5hmC detection, specifically targeting the Diff condition.

### 2.2. 5mC in Polycomb Subunits Is Differentially Enriched in Diff vs. Sphere HepaRG Culture

To assess regulatory targeting, DMLs were analyzed using the KYCG framework. Specifically, we studied the enrichment of DMLs in Transcription Factor Binding Sites (TFBS; ENCODE/ReMAP), as well as within chromatin state (Roadmap Epigenomics, ChromHMM), histone modification, and CpG island (CGI) annotations. This analysis was conducted separately for the 5mC DMLs that were significantly higher in Diff and in Spheres.

**Figure 2:**
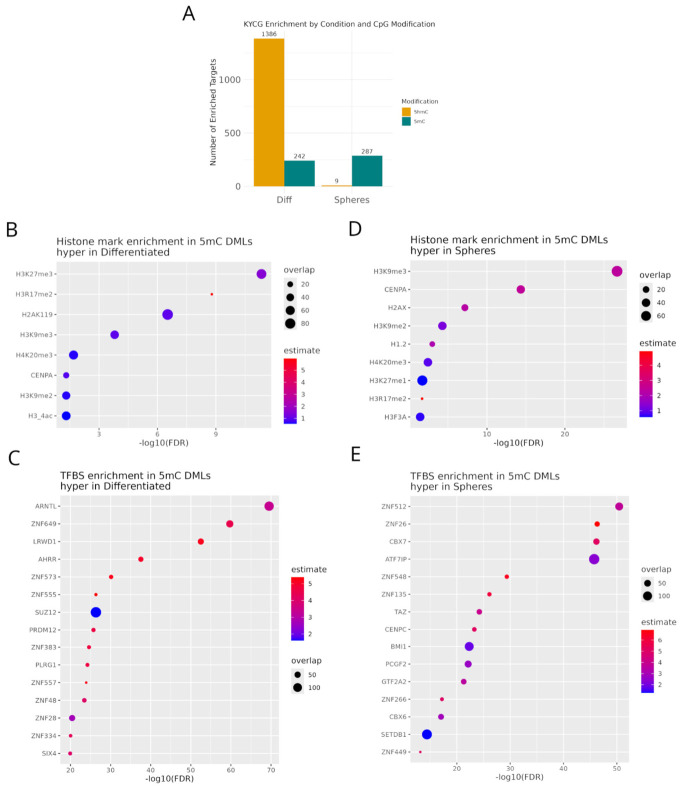
Regulatory annotation enrichment of differential 5mC CpGs in HepaRG models. 5mC KYCG enrichment analyses were performed separately in CpG sites hypermethylated in Differentiated (**left panels**) or Sphere (**right panels**) conditions. (**A**) Number of KYCG-enriched terms in Diff and Spheres combining different regulatory annotations (i.e., Transcription Factor Binding Sites, Chromatin states, Histone modifications, and CpG island annotations) and shown separately for 5mC and 5hmC. Top enriched 5mC histone modification terms are shown separately for Diff (**B**) and Spheres (**D**). Top enriched 5mC TFBS targets are shown separately for Diff (**C**) and Spheres (**E**). Overlap indicates the number of regions (5hmC DML regions) that overlap a given feature (TFBS or histone modifications), while Estimate reflects the effect size of enrichment.

First, we found a similar number of 5mC-enriched features between Diff and Sphere HepaRG models (242 and 287 targets, respectively) (Figure 2A). Diff hypermethylation heavily targets CGIs and, to a lesser degree, those regions upstream to CGIs (Shelves and Shores) (Table S3).

Moreover, differentiation-associated hypermethylation is characterized by a strong Polycomb Repressive Complex 2 (PRC2) signature, as illustrated by the deposited repressive mark H3K27me3 (Figure 2B), and the enrichment of the PRC2 subunits SUZ12 and EZH2 (Figure 2C and Table S3). Structurally, it distinctly targets bivalent transcription start sites (see target ‘10_TssBiv’ in Table S3, which corresponds to the enrichment in bivalent transcription start sites).

While HepaRG-Sphere hypermethylation also targets Islands and Shores, instead of H3K27me3, it is highly enriched in H3K9me3 (Figure 2D). In line with this, we found an enrichment in other heterochromatin elements like 9_Het and the methyltransferase SETDB1, alongside bivalent enhancers (12_EnhBiv) and bivalent flanking regions (11_BivFlnk) (Figure 2E, Table S4). The pattern of polycomb enrichment is also different in Spheres. Instead of PRC2, Sphere hypermethylation significantly targets Polycomb Repressive Complex 1 (PRC1) components, specifically CBX7, BMI1, PCGF2, and RING1 (Figure 2E, Table S4).

These findings suggest that Polycomb subunits are differentially assembled in these two HepaRG fate trajectories. Instead of PRC2/H3K27me3 accumulation seen in standard differentiation, Sphere formation is associated with reinforcement of repressive methylation programs targeting PRC1/H3K9me3.

### 2.3. Hepatocyte TFs Are Dynamically Modulated by 5hmC in Differentiation Conditions

**Figure 3:**
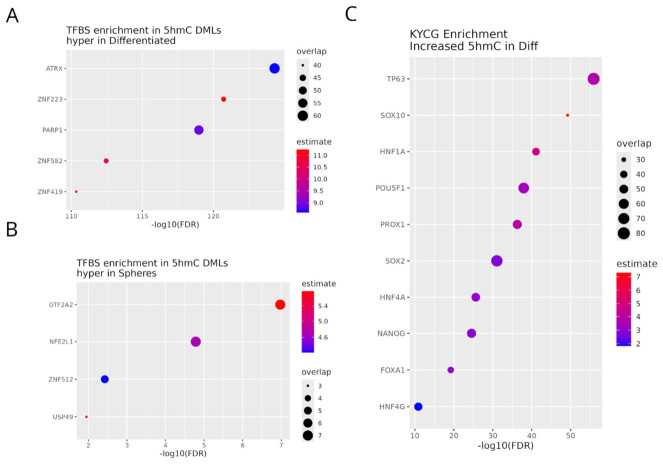
Regulatory annotation enrichment of differential 5hmC CpGs in HepaRG models. 5hmC KYCG TFBS enrichment analyses were performed separately in CpG sites hypermethylated in Diff (**A**) or Sphere (**B**) conditions. (**C**) KYCG enrichment in differentiation TFBS for 5hmC in the Differentiated condition. Overlap indicates the number of regions (5hmC DML regions) that overlap a given feature (TFBS), while Estimate reflects the effect size of enrichment.

KYCG analysis of 5hmC revealed a strong 5hmC asymmetry with 1386 5hmC-enriched features in Diff and only 9 in Spheres (Figure 2A). This is not surprising, considering the larger number of DMLs identified in Diff, as described above (see Figure 1C). Importantly, the Diff environment drives larger deposition of 5hmC across hundreds of targets (Figure 2A). Some of the top-most enriched targets included heterochromatin marks (H3K9me3 and H4K20me3) and targets of ATRX, PARP1, and a diversity of zinc finger proteins (Figure 3A and Table S5). In contrast, 5hmC enrichment in Spheres is extremely restricted (Table S6). It targets active enhancers (7_Enh), the PRC1-associated mark H2AK119ub, deep heterochromatin marks (H3K9me3, H4K20me3), and a handful of specific factors such as GTF2A2, NFE2L1, ZNF512, and USP49 (Figure 3B and Table S6).

Next, we specifically looked at TFs associated with hepatocyte differentiation. Among KYCG enrichment terms, we identified several classic markers of cell fate, with their cytosine modification dynamics differing depending on the culture condition. While this was not the case for 5mC, 5hmC enrichment was found to map significantly to targets of the master stem cell transcription factors POU5F1 (OCT4), NANOG, and SOX2 found in the Diff condition but not in Spheres (Table S5). Other differentiation factors, such as SOX10 and TP63, gain 5hmC in Diff and 5mC in Spheres (Figure 3C). When isolating master TFs that govern liver lineage commitment, we found a strong bias toward 5hmC hypermethylation in Diff conditions. This included core liver factors (i.e., HNF1A, HNF4A, HNF4G, FOXA1, and FOXA2) along with the hepatoblast specification factor PROX1, all of their targets enriched in 5hmC in Diff (Figure 3C). Conversely, the Spheres environment transitions to permanently methylating some of these exact same loci (i.e., HNF1A and PROX1) (Table S4). Of note, targets of AHRR, crucial for mature hepatocyte drug metabolism, were dually enriched in Diff, gaining both 5mC and 5hmC (Tables S3 and S5). In addition, NFE2L1 (critical for lipid homeostasis and stress response in the liver) is one of the only factors that gains 5hmC in Spheres (Figure 3B), though it also accumulates 5mC in Spheres and 5hmC in Diff (Tables S4 and S5).

Globally, 5hmC appears to have a dynamic role specific to Diff culture and including the regulation of hepatocyte-specific TFs. In contrast, Spheres culture may more permanently silence a subset of progenitor genes.

### 2.4. TF Activity Inferred from 5mC Aggregation

**Figure 4:**
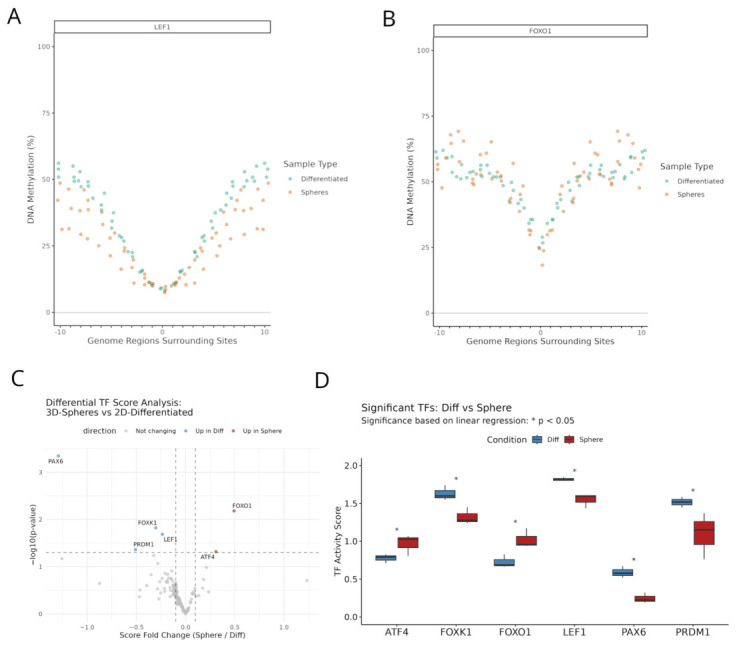
Activity of Transcription factors inferred from the 5mC landscape. Representative MIRA profiles for LEF1 (**A**) and FOXO1 (**B**) activities, with Diff samples shown in green and Spheres shown in red. (**C**) Differential MIRA score analysis represented as a volcano plot of fold change (x-axis) and *p*-value (y-axis). Fold changes correspond to the Spheres vs. Diff comparison (i.e., Spheres/Diff). The vertical dashed lines indicate the thresholds for biological effect size, set at a mean score difference of +/− 0.1 (Sphere − Diff). The horizontal dashed line represents the threshold for statistical significance, set at *p* = 0.05. Features in the top-left quadrant represent transcription factors significantly enriched in the ‘Diff’ condition, while those in the top-right quadrant are significantly enriched in the ‘Sphere’ condition. (**D**) MIRA score boxplots of TF activities for those TFs differentially significant in the Spheres vs. Diff comparison.

Finally, we studied TF activity inferred from 5mC data using MIRA, an algorithm that infers activity by looking at localized “dips” in 5mC methylation footprints from aggregated TF target regions [11] (Figure 4A,B). The shape of those “dips” is transformed into a score, reducing the DNA methylation profile to a number that predicts regulatory activity. We calculated such activity scores for 138 high-confidence human TFs in each of our samples. Next, we compared the scores between the two conditions using a linear model. We identified only six TFs with significant regulatory differences (linear regression *p* < 0.05) in inferred activity between the two conditions: PAX6, FOXO1, FOXK1, LEF1, PRDM1, and ATF4 (Figure 4A–D and Table S7). Among these, FOXO1 and ATF4 display a higher inferred activity in Spheres, while PAX6, FOXK1, LEF1, and PRDM1 show a higher activity in Diff (Figure 4C,D).

Functional over-representation analysis using all tested TFs as background did not identify significant enrichment of these differential TFs in any biological pathways. However, they are largely involved in the regulation of cell fate and differentiation (PAX6, PRDM1, LEF1, and FOXO1) and metabolic homeostasis (FOXO1, FOXK1, and ATF4).

Of note, these TFs were not among the top enrichments identified with KYCG. However, all six of them appear also abundant in 5hmC-enriched regions in the Diff condition, indicating that the altered 5mC footprints detected by MIRA may be linked to active DNA demethylation driven by the Diff culture environment.

## 3. Discussion

In this study, we demonstrate that the inherent sparsity of shallow long-read sequencing can be at least partially circumvented through functional aggregation (e.g., KYCG and MIRA), allowing for the extraction of global regulatory patterns from single-molecule modification data. By mapping 5mC and 5hmC dynamics in HepaRG cells, we reveal that Diff maturation and Sphere retrodifferentiation are not merely opposing bidirectional trajectories but are governed by fundamentally asymmetric epigenetic programs.

In addition to a switch in Polycomb composition and activity (based on histone mark enrichment), we identify differentiation as a process potentially driven by TET-mediated 5hmC targeted specifically toward established heterochromatin (H3K9me3, H4K20me3). The paradoxical concurrent enrichment of 5mC and 5hmC at these identical domains indicates a dynamic state of epigenetic turnover. We propose a model for this transition, where bulk 5mC globally consolidates the repression of alternative progenitor lineages, while localized 5hmC physically uncouples specific hepatic enhancers.

As shown by our analysis of 5mC in TFBS using MIRA, a limited number of TFs may drive regional chromatin opening and execute their TF program. Indeed, three of the TFs with higher activity in HepaRG-Diff are linked to cell differentiation: PAX6 is a master developmental regulator, LEF1 is a classic regulator of stem cell proliferation involved in hepatocyte heterogeneity by interacting with HNF4A (liver zonation) [13], and PRDM1 (also known as BLIMP1) is a critical repressor that drives the terminal differentiation of various cell types [14]. Although this does not necessarily indicate repression, epigenetic remodeling (5hmC accumulation) and increased activity of these factors in HepaRG-Diff point toward a dynamic regulation of stemness in these culture conditions.

Conversely, Sphere retrodifferentiation may rely on active epigenetic consolidation to maintain a progenitor-like state. The spheroid niche utilizes targeted de novo 5mC deposition to directly silence the adult hepatic master regulator *HNF1A*. Moreover, the unique 5mC hypermethylation at Polycomb Repressive Complex (PRC1/2) binding sites (*CBX7*, *BMI1*, *PCGF2*) suggests a more permanent silencing through DNA methylation. In addition, the exceptions to such silencing may be revealing. Indeed, the only two TFs with higher activity in Spheres, FOXO1 and ATF4, are master regulators of cellular stress and metabolism. In liver biology, FOXO1 regulates gluconeogenesis and insulin signaling [15], while ATF4 orchestrates the response to endoplasmic reticulum (ER) stress and amino acid deprivation [16]. Their significantly increased activity suggests that Spheres cultured cells are undergoing metabolic rewiring and stress-response adaptations compared to Diff conditions.

In this sense, the epigenetic trajectories identified here may add a molecular basis for the chemoresistance associated with Spheres HCC retrodifferentiation [3]. The Spheres use targeted 5mC hypermethylation to silence mature hepatic networks (e.g., HNF1A) and consolidate a Polycomb-driven stem-like state, a recognized hallmark of therapeutic evasion [2,17,18]. Beyond the 5mC-mediated silencing of mature xenobiotic metabolic pathways, Spheres may use focal 5mC/5hmC dual-marking at a few critical stress-sensor loci (e.g., *NFE2L1*) [19]. This configuration may confer retrodifferentiated cells with the transcriptional agility required to survive the proteotoxic and hypoxic stresses of the tumor microenvironment, highlighting chromatin plasticity as a mechanism of oncogenic resilience.

A limitation of the study is the lack of orthogonal validation. Although global epigenetic divergence is expected in these two phenotypically different trajectories, the specific changes identified with our pipeline will benefit from future comparison with other methods or extended testing in different biological contexts where shallow sequencing analyses may be of interest. Specific DMLs, TFs and markers identified here should be validated using techniques such as bisulfite-specific PCR, RT-qPCR, or chromatin immunoprecipitation assays. In addition, downsampling from deep sequencing datasets will provide a better estimation of specificity and sensitivity, as has been recently shown for short-read sequencing [20].

## 4. Methods

### 4.1. Cell Culture and Experimental Design

HepaRG cells were cultured in William’s media enriched with 10% Fetal calf serum clone II, 1% penstrep, L-glutamine (2 mM), insulin (5 µg/mL), and hydrocortisone (25 µg/mL). Proliferative HepaRGs were taken before reaching 50% confluence and cultured under two biological conditions in independent triplicates: (i) Differentiated enriched HepaRG hepatocytes (HepaRG-Diff) and (ii) Sphere-derived retrodifferentiated cells (HepaRG-Spheres). Differentiation and sphere formation were performed as previously described [3], including an enrichment in hepatocyte HepaRG [4]. Genomic DNA was extracted from each replicate using the PureLink Genomic DNA extraction kit (ThermoFisher, Waltham, MA USA) according to the manufacturer’s instructions at matched endpoints for epigenetic profiling.

### 4.2. Nanopore Sequencing and Base Modification Calling

Native DNA methylation profiling was performed using Oxford Nanopore long-read sequencing in a single flowcell (FLO-MIN106 flow cells with pore 9.4.1 chemistry) with the SQK-RBK004 Rapid Barcoding Kit according to the manufacturer’s instructions (Oxford Nanopore Technology, Oxford, UK), sequenced for 72 h using the MinION device (Oxford Nanopore Technology, Oxford, UK). Raw signal-level data were basecalled using Guppy version 6.1.7, and cytosine modifications (5mC and 5hmC) were detected using the Remora model (ont-remore version 1.0.0, dna_r9.4.1_450bps_fast.cfg), enabling simultaneous identification of both modifications on native DNA. Reads were aligned to the human reference genome (hg38). Per-CpG methylation and 5hmC frequencies were extracted and aggregated across reads to generate locus-level modification proportions for each biological replicate. Given the shallow genome-wide sequencing depth inherent to long-read modification detection, analyses were restricted to CpGs that were measurable (1x) across at least two biological replicates per condition. This filtering yielded approximately 171,000 CpGs suitable for testing across samples. Coverage distributions and CpG retention statistics are provided in Figure 1.

### 4.3. Differential Methylation Analysis

Differentially methylated loci (DMLs) were identified using the R/Bioconductor package Dispersion Shrinkage for Sequencing Data (DSS version 2.60.0) [9], which models count-based methylation proportions using a beta-binomial framework. Pairwise comparisons between HepaRG -Diff and HepaRG-Spheres were performed separately for 5mC and 5hmC using biological triplicates per condition. Smoothing was enabled to stabilize variance estimates across neighboring CpGs. To balance statistical power and specificity under sparse coverage, DMLs were defined using a *p*-value below 0.05 and a delta threshold of at least 10% between conditions. This threshold was selected to provide sufficient foreground size for regulatory enrichment analyses while avoiding excessive inclusion of marginal-effect CpGs. Although differentially methylated regions (DMRs) were initially identified as clusters of ≥3 significant DMLs within 300 bp and a minimum length of 50 bp, subsequent regulatory interpretation focused primarily on single-CpG–based analyses due to sparse regional CpG density.

### 4.4. KYCG Enrichment Analysis

To assess whether DMLs preferentially localized within specific regulatory annotations, we applied the KnowYourCG (KYCG version 1.8.0) framework [10]. KYCG evaluates enrichment of CpG sets against curated databases using hypergeometric testing relative to a defined CpG universe. For enrichment testing, each DML was expanded into a ±50 bp window to increase sensitivity for motif-level overlap. The foreground consisted of DML-centered CpGs, with the background universe comprising all CpGs passing coverage filtering (~171k sites). CpG coordinates were converted into Yet Another Methylation Encoder (YAME)-compressed format for efficient intersection against curated knowledgebases, which included Transcription Factor Binding Sites (TFBS; ENCODE/ReMAP), Chromatin state annotations (Roadmap Epigenomics, ChromHMM), Histone modification datasets, and CpG island annotations. Statistical significance was determined using hypergeometric testing with Benjamini–Hochberg False Discovery Rate (FDR) correction. Enrichments were evaluated separately for hypermethylated and hypomethylated DMLs in each condition and for both 5mC and 5hmC datasets.

### 4.5. Regional Regulatory Activity Inference (MIRA)

To determine whether focal CpG-level perturbations translated into coordinated regional accessibility changes, we applied Methylation-Based Inference of Regulatory Activity (MIRA version 1.34.0) [11]. MIRA aggregates methylation profiles across sets of genomic regions (e.g., TFBS instances) and quantifies local methylation depletion relative to flanking regions as a proxy for regulatory accessibility. We extended CpG sites by 2000 bp upstream and downstream and aggregated methylation profiles in those regions, using a 21-bin parameter with a minimum base coverage per bin of one. MIRA scores were computed for each replicate, and differences between conditions were evaluated using linear regression models. We calculated MIRA activity scores for 138 high-confidence human TFs.

### 4.6. Genomic Annotation

Genomic features were annotated using the TxDb.Hsapiens.UCSC.hg38.knownGene package. CpGs and DMLs were assigned to promoters, gene bodies, intergenic regions, and CpG island–related features (islands, shores, and shelves) using standard Bioconductor workflows.

## 5. Conclusions

Using a combination of recent bioinformatic algorithms, it is possible to extract functional 5mC/5hmC information from shallow long-read sequencing. Techniques such as smoothing (DSS), aggregation (KYCG) and binning (MIRA) provided a global view of two contrasting pathways of in vitro differentiation. We could detect how 5mC/5hmC turnover may have a stronger impact in refining differentiation, how polycomb machinery may be coerced towards consolidation and definitive silencing in Spheres, and how prevailing TF activity may point to the source of chemoresistance in retrodifferentiation. More broadly, hepatic plasticity does not seem governed by static on/off states but by highly dynamic zones of coupled methylation-demethylation cycling.

## Data Availability

The data supporting the findings of this study have been deposited in the Zenodo repository and are available under DOI: https://doi.org/10.5281/zenodo.20457653, accessed on 29 May 2026.

## Supplementary Materials

The following supporting information can be downloaded at: https://www.mdpi.com/article/10.3390/epigenomes10020036/s1, Table S1. 5mC DMLs in Differentiation versus Spheres. Table S2. 5hmC DMLs in Differentiation versus Spheres. Table S3. 5mC KYCG enrichments in Differentiation (relative to Spheres). Table S4. 5mC KYCG enrichments in Spheres (relative to Differentiation). Table S5. 5hmC KYCG enrichments in Differentiation (relative to Spheres). Table S6. 5hmC KYCG enrichments in Spheres (relative to Differentiation). Table S7. MIRA scores, linear regression results (Spheres vs. Differentiation).
